# Could current factors be associated with retrospective sports injuries in Brazilian jiu-jitsu? A cross-sectional study

**DOI:** 10.1186/s13102-017-0080-2

**Published:** 2017-10-23

**Authors:** Dayana das Graças, Letícia Nakamura, Fernando Sérgio Silva Barbosa, Paula Felippe Martinez, Filipe Abdalla Reis, Silvio Assis de Oliveira-Junior

**Affiliations:** 1 Postgraduate Program in Health and Development, Federal University of Mato Grosso do Sul, Campo Grande, MS Brazil; 2 School of Physical Therapy, Federal University of Mato Grosso do Sul, Campo Grande, MS Brazil; 3 School of Physical Therapy, Anhanguera University – UNIDERP, Campo Grande, MS Brazil; 4 Laboratoty of Striated Muscle Study (LEME/ CEI), Federal University of Mato Grosso do Sul, Campo Grande, MS Brazil

**Keywords:** Epidemiology, Athletic performance, Athletic injuries, Sports medicine

## Abstract

**Background:**

Brazilian jiu-jitsu is characterized by musculoskeletal disorders and high occurrence of sports injuries. The present study was aimed to analyze some internal factors, as well as to describe occurrence and characteristics of retrospective musculoskeletal injuries in different age groups of Brazilian jiu-jitsu practitioners.

**Methods:**

One hundred ninety-three Brazilian jiu-jitsu practitioners, which were divided into three age groups: Adolescent, Adult, and Master. Besides anthropometric characterization, standard clinical tests were conducted to analyze the global and segmental joint flexibility, lumbar spine range of motion, and handgrip strength. Sports injury occurrence and total physical activity were obtained from an adapted morbidity survey and International Physical Activity Questionnaire - Short Form (IPAQ-SF), respectively.

**Results:**

A total of 247 cases of retrospective injuries was registered (1.27 injury/ participant). Occurrence of rectus femoral muscle retraction in the right leg was increased within Master. Adult and Master have exhibited higher occurrence of sports injuries than Adolescent group (*p* < 0.05). Joint injuries were the most common sports-related injuries by all Brazilian jiu-jitsu practitioners. While female gender and exposure time constituted the most predictive variables for sports injury occurrence in Adolescent, graduation level was more associated with sports injuries occurrence in Adult.

**Conclusions:**

Joint injuries derived from combat demands were the main sports injury in all age categories of Brazilian jiu-jitsu. Master subjects presented a higher occurrence of clinical changes and retrospective musculoskeletal injuries in relation to other age groups. Female gender and exposure time constituted the main predictive factors in adolescent subjects, while graduation category was more directly associated with retrospective injury onset in the Adult group.

## Background

Brazilian jiu-jitsu is a self-defense sport based on the principle of defeating the adversary: subjugating them by using their own strength [1]. As a strategic sport, Brazilian jiu-jitsu is a high-intensity combat sport, combining intermittent bouts of very intense anaerobic exercise, interspersed with lower-intensity periods of aerobic activities [2–5]. It requires concentration, balance and important physical conditioning to execute takedowns, sweeps, joint locks and chokes in different circumstances of body combat [1–5].

These different requisites of Brazilian jiu-jitsu, as well as high sport competitiveness, excessive training intensity, and differences between stress and recovery have constituted external risk factors for the onset of musculoskeletal injuries in fighters [6–9]. Also, age, body composition, previous history of sports injuries, posture, and muscle flexibility are important internal characteristics, which have been associated with the occurrence of new injuries [1, 6, 9]. From this pathophysiological perspective, a complete understanding of the potential causes of sports injury needs to address the multifactorial nature of musculoskeletal injuries in any sport modality [6].

In this context, possible associations among different characteristics and musculoskeletal injuries occurrence have been continuously reported in many sport modalities, such as track and field [10], volleyball [11], soccer [12], and judo [13]. However, regarding Brazilian jiu-jitsu, relationships among different external and internal features with respect to the injuries occurrence still need to be clarified. It is noteworthy that the investigation of clinical predisposing factors and characterization of sports injuries are of great prophylactic potential [1].

Since different internal characteristics may be progressively affected by the age step [6, 14], the present study aimed to analyze some internal elements, such as flexibility, training exposure and anthropometric measures, as well as to describe occurrence and characteristics of retrospective musculoskeletal injuries in different age groups of Brazilian jiu-jitsu practitioners. Although these parameters have not been clarified in previous studies with respect to Brazilian jiu-jitsu practitioners, our initial hypothesis is that master practitioners, i.e. 30-year old practitioners or older, present more significant clinical disorders and increased occurrence of musculoskeletal injuries, when compared to younger participants. Based on different circumstances of combat [1–5], joint injuries in upper limbs are the main type of injury in all age group of Brazilian jiu-jitsu fighters.

## Methods

### Ethical approval and consent to participate

The protocol was approved by the local ethics committee (Protocol 575.773/ 2014; CAAE 26804614.7.0000.0021), and all participants were given verbal and written information before they agreed to participate and to sign a consent form. Parental or legal guardian consent, written and verbal, was obtained for all participants under the age of 18 years-old.

### Participants

This cross-sectional study was performed at the Laboratory of Striated Muscle Study (LEME/ CEI) Federal University of Mato Grosso do Sul, Campo Grande, MS, Brazil. In order to select participants, we chose to use a convenience sample, which means subjects of both sexes, aged over 12 years old and in regular training practice for at least 12 months. At the moment of start of the present study, the population of Brazilian jiu-jitsu fighters from Campo Grande, MS, Brazil, was constituted by 345 athletes. In order to determine the minimum size sample to the development of the investigation, the measure for prevalence of injured subjects was considered to be 50%. Based on this, and take into account a level of significance of 5%, as well as population dimension, minimum sample size totalized 183 subjects. The studied sample was composed of 193 subjects from 13 Brazilian jiu-jitsu training sites in Campo Grande, MS, Brazil.

### Anthropometric characterization and design of groups

Participants were instructed to wear minimum clothes for all assessments. They were also instructed to refrain from exercise on the day of the tests. Height and body weight measures were obtained, according to the proceedings of previous studies [14, 15]. Subjects constituted different levels and categories, were 24.5 ± 9.1 years old, and presented 74.4 ± 19.0 kg of body weight, 170.0 ± 10.0 cm of height, and 37.0 ± 41.1 months of regular practice of Brazilian jiu-jitsu.

The study design involved subjects being distributed into three age groups [16]: **Adolescents**, with adolescents practitioners aged over 12 up until 17 years old; **Adults**, constituted by adult subjects, aged between 18 and 30 years old; **Masters**, integrated by master subjects, aged over 30 years old.

### Sports injuries and physical activity analyses

The participants were interviewed and evaluated only once to obtain information about history of practice, level of physical activity, and musculoskeletal injury occurrence due to Brazilian jiu-jitsu practice. The International Physical Activity Questionnaire - Short Form (IPAQ-SF) was used to determine the total physical activity of each participant. Taking into account the score of physical activity, subjects were classified into sedentary, insufficiently active, active, and very active [17].

In order to obtain information about retrospective sports injuries derived from Brazilian jiu-jitsu practice, we used an adapted morbidity survey [18]. The present device was adjusted from previous purposes [14, 15] and integrated information about injury occurrence, besides nature, etiological mechanisms, anatomical location, and severity. Sport injury was defined as any symptomatic manifestation of pain or physical dysfunction, due to training practice or Brazilian jiu-jitsu competitions, resulting in training alterations, whether in form, duration, intensity or frequency [19], and integrated cases with onset until 4 years of regular practice of Brazilian jiu-jitsu training. All administering of surveys/ questionnaires and recording of information were performed by two trained researchers. This began with explanations on the objectives and development of the study, followed by implementation of the survey and recording of the notes on the survey.

Afterwards, clinical tests were conducted to analyze the global and segmental joint flexibility, lumbar spine range of motion, and handgrip strength.

### Clinical test

**Fig. 1:**
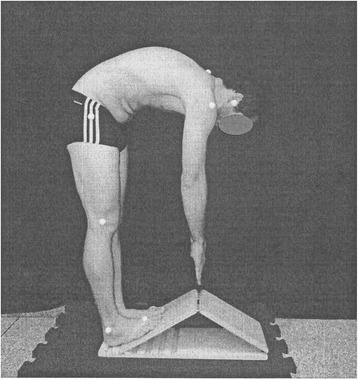
Posture 1; analysis of global alignment of the posterior static muscle chain

To analyze global joint flexibility, we considered methodological principles described by Kendall et al. [20]. The posterior static muscle chain is constituted by diverse muscles from paravertebral, lumbar, gluteus, and posterior leg regions. In order to investigate the global alignment of the posterior static muscle chain, each participant stood on a ramp with 30° tilt, maintaining 60° of range of flexion to hip and knee joints (Posture 1; Fig. 1). Altered global flexibility of this muscle chain was considered from occurrence of accentuated lumbar lordosis, horizontal pelvis inclination, reduced range of knee flexion, or cavovarus feet [20].

**Fig. 2:**
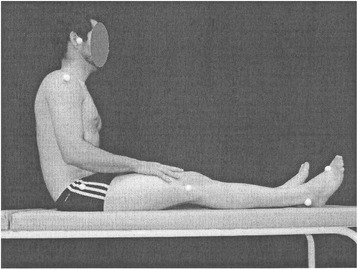
Posture 2; analysis of global alignment of the anterior static muscle chain

The anterior static muscle chain, which is composed of scalene, psoas, intercostal, diaphragm, and anterior leg muscles, was also assessed and each participant was required to maintain a sitting position on a bed with the legs extended (Posture 2; Fig. 2). Retraction of the anterior static muscle chain was confirmed from anterior positioning of the head and/or occurrence of pelvic retroversion [20]. Posture images were obtained using a photographic chamber (Sony, model DSC-TF1, Brazil), one plumb line, and cutaneous markers [21]. Images underwent independent assessment by three examiners.

The mobility of back and flexibility of leg muscles were analyzed with the use of the sit-and-reach test (Wells and Dillon’s Bench) [22]. The subjects sat with their feet approximately hip-wide against the testing box. They kept their knees extended and placed the right hand over the left, and slowly reached forward as far as they could by sliding their hands along the measuring board. For the present assessment, the test was conducted three times with an interval of 30 to 40 s between tests [22, 23].

The Modified Schober’s Test was carried out to investigate the lumbar spine range of motion, according to proceedings previously published [24]. A mark was placed 5 cm below and 10 cm above the lumbosacral junction. The participant was asked to bend forward as far as possible and the stretched distance of these two points was measured as the result of the test.

The Thomas test was used to assess bilateral rectus femoris muscle flexibility in each participant [25]; for this, a fleximeter (Code®, Brazil) was attached to tibia midpoint. The subject was placed in a supine position with the knees bent over the edge of the examination table, and was instructed to flex one knee to the chest and hold it. At the same time, the angle of the test knee (opposite of the knee held to the chest) was to remain at 90°, and the hip and posterior thigh of the test leg was to remain in a stationary position and regular against the examination table. The muscle flexibility was scored as *normal* if the tested knee remained in a stationary 90° position. Differentially, the presence of muscle retraction was confirmed if the test knee extended and moved to a position higher than 90°. Both legs were assessed.

Maximal isometric handgrip strength was recorded using a handheld handgrip dynamometer (Saehan®, Smedley-Type, Korea), according to the guidelines of the American Society of Hand Therapists [26]. The device measures strength in kilograms, with a precision of 0.1 kg. Participants were instructed to sit in a straight-backed chair with feet placed flat on the floor, shoulder adducted and neutrally rotated, elbow flexed at 90°, and the forearm and wrist in neutral position. Grip strength was measured 3 times, and the rest between measurements was 15 s. The participants were instructed to apply maximal power for 3 s and work at maximal effort in every trial; maximal value of handgrip strength obtained among the three repetitions was considered as result [26]. An allometric scaling of grip strength by body weight was adopted to obtain results [27].

### Statistical analysis

Demographic and anthropometric variables, history and training period, back mobility, and maximal isometric handgrip strength results were analyzed by One-Way ANOVA. When significant differences were found (*p* < 0.05), the post hoc Student-Newman-Keuls’ multiple comparisons test for parametric or Dunn’s test for non-parametric distributions were performed. Regarding the qualitative variables, sports injuries results, muscle retraction occurrence, and categories of physical activity were analyzed using Goodman’s test for contrasts between and within multinomial populations. Moreover, categorical and continuous data were analyzed using binary logistic regression in software Systat 13 (SYSTAT, Chicago, Illinois). For all internal characteristics, odds ratios (OR) and 95% confidence intervals (CI) were calculated, and level of significance was considered to be 5%.

## Results

With respect to the constitution of the age groups, all groups have showed a greater proportion of male in relation to female subjects (Table 1); Masters integrated higher proportion of male subjects, in comparison to the Adolescents. Relative to graduation, Adolescents presented greater frequency of white belt rank when compared to Adults and Masters; adults and master categories exhibited similar proportions of intermediate and black belt ranks (Table 1).

**Table 1:** Constitution of the age groups according gender and graduation

Variables		Groups		
		G1 (Adolescents)	G2 (Adults)	G3 (Master)
Gender	Female	16 (26.2%)^Ba^	14 (17.7%)^ABa^	5 (9.4%)^Aa^
	Male	45 (73.8%)^Ab^	65 (82.3%)^ABb^	48 (90.6%)^Bb^
Belt rank (Graduation)	White	52 (85.2%)^Bc^	38 (48.1%)^Ab^	16 (30.2%)^Ab^
	Other	9 (14.8%)^Ab^	39 (49.4%)^Bb^	29 (54.7%)^Bab^
	Black	0 (0.0%)^Aa^	2 (2.5%)^ABa^	8 (15.1%)^Ba^
Participants (n)		61	79	53

^A,B^ *p* < 0.05 to horizontal comparisons, between different groups; ^a, b, c^ *p* < 0.05 to vertical comparisons, within group. Goodman’s test for contrasts between and within multinomial populations

Additionally, other characteristics and musculoskeletal injury occurrence are presented in Table 2. Adults and Masters groups showed increased measures of body weight, height, time of practice, and maximal isometric handgrip strength compared to the Adolescents; moreover, Masters presented higher values of body weight, time of practice, and maximal isometric handgrip strength than Adults. In addition, mobility of back and flexibility of leg muscles, analyzed by the sit-and-reach test, were similar among age groups (*p* = 0.08). When only subjects with one or more occurrences of sports injuries were considered, Master participants presented lower results from sit-and-reach test in comparison to the Brazilian jiu-jitsu adolescent practitioners (Adolescents 25.80 ± 7.24, Adults 26.66 ± 8.71, Masters 23.72 ± 7.75 cm; *p* = 0.004). Concerning sports injury occurrence, 247 cases of injuries were registered, resulting in 1.27 case/ subject. Among age groups, 108 (43.72%) cases of injury were reported by Adults; however, the indexes of injury occurrence were increased in Masters, followed by Adults, and Adolescents (Table 2).

**Table 2:** General characteristics and occurrence of musculoskeletal injuries in Brazilian jiu-jitsu practitioners

Variables	Groups		
	G1 (Adolescents)	G2 (Adults)	G3 (Master)
Age (years)	15.0 (12.0 — 15.0)	25.0 (22.0 — 27.0)^*^	35.0 (32.8 — 38.0)^*, #^
Height (cm)	1.64 ± 0.11	1.74 ± 0.08^*^	1.75 ± 0.07^*^
Body weight (kg)	58.1 ± 13.6	76.6 ± 12.9^*^	89.9 ± 17.4 ^*, #^
TP (months)	12.0 (12.0 — 13.0)	24.0 (12.0 — 48.0)^*^	36.0 (12.0 — 99.0)^*, #^
Exposure time (h)	8.00 (4.38 — 12.00)	5.00 (4.00 — 8.00)^*^	5.00 (4.00 — 7.30)^*^
Sit-and-reach test (cm)	26.4 ± 6.5	26.5 ± 9.0	23.7 ± 7.8
Hand-grip strenght (kgf)	27.5 ± 11.1	38.3 ± 11.3^*^	45.8 ± 11.0^*, #^
Injuries/ Practitioner	0.59	1.37	1.94
Injuries/ I. Practitioner	1.24	1.74	2.19
Injuries (cases)	36	108	103

*TP* time of practice, *Flexibility* flexibility of back and leg muscles, *Hand-grip strenght* maximal isometric handgrip strenght, *Injuries/I. Practitioner injury incidence per injured practitioner*. Height, body weight, flexibility, and hand-grip strenght results are expressed in mean ± standard deviation, ANOVA and Student-Newman-Keuls test. Age, time of practice, and exposure time results are presented in median and interval between 25th and 75th percentile, ANOVA and Dunn test; ^*^ *p* < 0.05 vs. G1; ^#^ *p* < 0.05 vs. G2

Results of muscle flexibility, lumbar spine range of motion, and physical activity levels are presented in Table 3. Concerning the segmental muscle flexibility, the prevalence of rectus femoris muscle retraction in the right leg was increased within Masters. Moreover, the proportion of cases with normal lumbar spine range of motion was higher than the prevalence of cases with reduced lumbar movement in all age groups. Regarding global alignment of the posterior static muscle chain, Adult subjects presented a higher proportion of muscle retraction than Adolescent practitioners. The anterior static muscle chain (Posture 2) as well as physical activity levels were unaffected by the age category; the proportion of subjects with normal alignment of the anterior static muscle chain and very active status for physical activity were amplified within all groups (Table 3).

**Table 3:** Muscle retractions, lumbar spine range of motion, and physical activity classification

Variables	Responses		Groups			Total
			G1	G2	G3	
Right Leg	MHF	Absent (−)	32 (52.5)^Aa^	39 (53.4)^Aa^	28 (47.5)^Aa^	99
		Present (+)	29 (47.5)^Aa^	34 (46.6)^Aa^	31 (52.5)^Aa^	94
	RF	Absent (−)	35 (57.4)^Aa^	31 (42.5)^Aa^	22 (37.3)^Aa^	88
		Present (+)	26 (42.6)^Aa^	42 (57.5)^Aa^	37 (62.7)^Ab^	105
Left Leg	MHF	Absent (−)	33 (54.1)^Aa^	36 (49.3)^Aa^	26 (44.1)^Aa^	95
		Present (+)	28 (45.9)^Aa^	37 (50.7)^Aa^	33 (55.9)^Aa^	98
	RF	Absent (−)	32 (52.5)^Aa^	35 (47.9)^Aa^	28 (47.5)^Aa^	95
		Present (+)	29 (47.5)^Aa^	38 (52.1)^Aa^	31 (52.5)^Aa^	98
Schober		Absent (−)	54 (88.5)^Ab^	63 (86.3)^Ab^	51 (86.4)^Ab^	168
		Present (+)	7 (11.5)^Aa^	10 (13.7)^Aa^	8 (13.6)^Aa^	25
Posture 1		Absent (−)	30 (49.2)^Ba^	19 (26.0)^Aa^	17 (28.8)^ABa^	66
		Present (+)	31 (50.8)^Aa^	54 (74.0)^Bb^	42 (71.2)^ABb^	127
Posture 2		Absent (−)	47 (77.0)^Ab^	60 (82.2)^Ab^	49 (83.1)^Ab^	156
		Present (+)	14 (23.0)^Aa^	13 (17.8)^Aa^	10 (16.9)^Aa^	37
Physical activity categories		Sedentary	7 (11,5)^Aab^	4 (5,5)^Aa^	0 (0,0)^Aa^	11
		Irreg. Active	1 (1,6)^Aa^	3 (4,1)^Aa^	4 (6,8)^Aab^	8
		Active	13 (21,3)^Ab^	7 (9,6)^Aa^	13 (22,0)^Ab^	33
		Very Active	40 (65,6)^Ac^	59 (80,8)^Ab^	42 (71,2)^Ac^	141

*MHF* monoarticular hip flexor flexibility, *RF* rectus femoris muscle flexibility, *Irreg. Active* irregularly active; ^A,B^ *p* < 0.05 to horizontal comparisons, between different groups; ^a,b^ *p* < 0.05 to vertical comparisons, within group. Goodman’s test for contrasts between and within multinomial populations

Epidemiological characterization of sports injuries in terms of nature (type), anatomical localization, and etiological mechanisms is presented in Table 4. Generally, Adults and Masters have exhibited higher occurrence of sports injuries than Adolescents group (*p* < 0.05). Concerning the type, joint hurts have integrated the most common sports-related injuries among Brazilian jiu-jitsu practitioners, which may be confirmed within all age groups. On the other hand, reports of muscle and joint injuries were proportionally similar within Adults and Masters, and muscle and tendon injuries have been more numerous in Masters compared to Adolescents (*p* < 0.05).

**Table 4:** Sports injuries according to nature (type), anatomical location, etiological mechanisms, and age group

Variables		Age group			Total
		G1	G2	G3	
Nature (type)	Muscle	4 (11.1)^Aa^	28 (25.9)^ABb^	40 (38.8)^Bb^	72 (29.0)
	Joint	29 (80.6)^Bb^	73 (67.6) ^Ac^	48 (46.6)^Ab^	150 (61.0)
	Bone	3 (8.3)^Aa^	3 (2.7)^Aa^	4 (3.9)^Aa^	10 (4.0)
	Tendon	0 (0.0)^Aa^	4 (3.7)^ABa^	11 (10.7)^Ba^	15 (6.0)
	Total	36 (14.6)	108 (43.7)	103 (41.7)	247
Location	Head/ Neck	0 (0.0)^Aa^	1 (0.9)^Aa^	5 (4.9)^Aa^	6 (2.4)
	Trunk	3 (8.3)^Aa^	13 (12.0)^Ab^	15 (14.6)^Aa^	31 (12.6)
	Upper Limbs	17 (47.2)^Ab^	51 (47.2)^Ac^	45 (43.7)^Ab^	113 (45.7)
	Lower Limbs	16 (44.4)^Ab^	43 (39.8)^Ac^	38 (36.9)^Ab^	97 (39.3)
	Total	36 (14.6)	108 (43.7)	103 (41.7)	247
Mechanism	Choke/ Arm Wrench	6 (16.7)^Aa^	18 (16.7)^Aab^	13 (12.6)^Aab^	37 (15.0)
	Takedown/ Attack	5 (13.9)^Aa^	26 (24.1)^Ab^	26 (25.2)^Ab^	57 (23.1)
	Sweep	1 (2.8)^Aa^	23 (21.3)^Bb^	21 (20.4)^Bb^	45 (18.2)
	Tumbling/ Trauma	21 (58.3)^Bb^	17 (15.7)^Aab^	20 (19.4)^Ab^	58 (23.5)
	Physical Training	2 (5.6)^Aa^	18 (16.7) ^Aab^	19 (18.4) ^Ab^	39 (15.8)
	Others	1 (2.8)^Aa^	6 (5.6)^Aa^	4 (3.9)^Aa^	11 (4.5)
	Total	36 (14.6)	108 (43.7)	103 (41.7)	247

^A, B^ *p* < 0.05 to horizontal comparisons, between different groups; ^a, b^ *p* < 0.05 to vertical comparisons, within group. Goodman’s test for contrasts between and within multinomial populations

Concerning location, upper and lower limbs constituted the main anatomical sites for onset of sports injuries in Brazilian jiu-jitsu, as sustained by all age groups. Moreover, circumstances of tumbling/ trauma were the most frequent etiological mechanisms for sports injuries occurrence among Adolescent practitioners, while sweep was more commonly referred as the etiological agent for injuries in Adult and Master practitioners. Within group, takedown/ attack situations were as related as sweep in the etiology of sports injuries among adult subjects. Takedown/ attack, sweep, tumbling/trauma, and physical training activities were similarly referred as causative agents of sports injuries within the Master group (Table 4).

Based on responses about nature (type), localization, and etiological mechanisms, Table 5 brings the descriptive distribution of the most common joint sports injuries among Brazilian jiu-jitsu practitioners. Injuries localized at shoulders constituted the most related record among all participants, integrating 44.3% of the joint responses (*n* = 50). Tumbling/ trauma circumstances were the primary mechanisms for occurrence of shoulder injuries, as supported by all age groups. Additionally, choke/ arm wrench constituted the primary mechanism for onset of elbow hurts in Adolescents and Masters, while takedown/attack was responsible for the most frequent handle/hands injuries in Adults and Masters (Table 5).

**Table 5:** Description of joint musculoskeletal injuries according etiological mechanisms, anatomical localization, and age group

Variables		Age group			Total
Mechanism	Localization	G1	G2	G3	
Choke/ Arm Wrench	Shoulder	2 (33.3)	5 (45.5)	2 (25.0)	9 (36.0)
	Elbow	4 (66.7)	4 (36.4)	5 (62.5)	13 (52.0)
	Wrist/ Hands	0 (0.0)	2 (18.2)	1 (12.5)	3 (12.0)
	Total	6	11	8	25
Takedown/ Attack	Shoulder	0 (0.0)	4 (19.0)	1 (6.7)	5 (12.8)
	Elbow	2 (66.7)	5 (23.8)	1 (6.7)	8 (20.5)
	Handle/ Hands	1 (33.3)	12 (57.1)	13 (86.7)	26 (66.7)
	Total	3	21	15	39
Sweep	Shoulder	0 (0.0)	4 (66.7)	5 (83.3)	9 (75.0)
	Elbow	0 (0.0)	2 (33.3)	0 (0.0)	2 (16.7)
	Handle/ Hands	0 (0.0)	0 (0.0)	1 (16.7)	1 (8.3)
	Total	0	6	6	12
Tumbling/ Trauma	Shoulder	4 (50.0)	5 (83.3)	7 (100.0)	16 (76.2)
	Elbow	2 (25.0)	1 (16.7)	0 (0.0)	3 (14.3)
	Handle/ Hands	2 (25.0)	0 (0.0)	0 (0.0)	2 (9.5)
	Total	8	6	7	21
Physical Training	Shoulder	0 (0.0)	5 (71.4)	6 (66.7)	11 (68.8)
	Elbow	0 (0.0)	2 (28.6)	3 (33.3)	5 (31.2)
	Handle/ Hands	0 (0.0)	0 (0.0)	0 (0.0)	0 (0.0)
	Total	0	7	9	16

In the pathophysiological aspect, and as support to the design of groups, age as well as global flexibility of the posterior static muscle chain constituted the most predictive variables for sports injury onset among Brazilian jiu-jitsu practitioners (*p* < 0.05); the binary logistic regression model revealed a statistical significance in predicting sports injury occurrence (*p* < 0.001; Table 6).

**Table 6:** Binary regression of injury risk factors in Brazilian jiu-jitsu practitioners

Variable	Coefficient	OR	Border values CI (95%)		*p*-value
Constant	−1.548	–	–	–	0.733
Age	−0.078	0.925	0.865	0.990	0.024*
Female sex	0.463	1.588	0.520	4.848	0.416
Height	3.014	20.368	0.154	2685.514	0.226
Body weight	−0.017	0.984	0.957	1.011	0.233
Time of practice	0.005	1.005	0.992	1.018	0.471
Exposure time	−0.041	0.960	0.876	1.052	0.381
RF, Left leg	0.003	1.003	0.958	1.051	0.883
RF, Right leg	−0.014	0.986	0.941	1.033	0.557
Hand-grip-strength, Dhg	−0.004	0.996	0.986	1.006	0.430
Hand-grip-strength, NDhg	0.002	1.002	0.992	1.013	0.647
Sit-and-reach test	0.010	1.010	0.961	1.062	0.703
Posture 1	1.189	3.283	1.452	7.421	0.004*
Posture 2	−0.537	0.584	0.214	1.593	0.294
Physical activity	−0.294	0.745	0.461	1.204	0.230
Graduation	−0.233	0.792	0.625	1.005	0.055
Schober	0.022	1.022	0.912	1.146	0.706

*OR* odds ratio, *RF* rectus femoris muscle flexibility, Hand-grip-strength, *Dhg* dominant hand-grip-strength, Hand-grip-strength, *NDhg* non-dominant hand-grip-strength, Flexibility: flexibility of back and leg muscles; ^*^ *p* < 0.05

Relative to each age group, female gender and exposure time constituted the most predictive variables for sports injury occurrence among Brazilian jiu-jitsu adolescent practitioners (*p* < 0.05); the binary logistic regression model revealed a statistical significance in predicting sports injury occurrence (*p* = 0.004; Table 7).

**Table 7:** Binary regression of injury risk factors in Brazilian jiu-jitsu adolescent practitioners

Variable	Coefficient	OR	Border values CI (95%)		*p*-value
Constant	−2.171	–	–	–	0.858
Age	−0.46	0.628	0.272	1.445	0.274
Female sex	5.34	209.34	2.014	21,759	0.024*
Height	8.995	8066	0.007	8.94	0.205
Body weight	0.044	1.045	0.949	1.152	0.371
Time of practice	−0.018	0.982	0.877	1.099	0.750
Exposure time	−0.363	0.696	0.504	0.961	0.028*
RF, Left leg	−0.001	0.999	0.906	1.102	0.990
RF, Right leg	0.031	1.032	0.906	1.174	0.638
Hand-grip-strength, Dhg	0.004	1.004	0.973	1.036	0.802
Hand-grip-strength, NDhg	−0.016	0.984	0.948	1.021	0.395
Sit-and-reach test	−0.186	0.830	0.601	1.145	0.257
Posture 1	−0.335	0.716	0.004	138.971	0.901
Posture2	1.327	3.770	0.007	24.550	0.273
Physical activity	2.218	9.191	0.594	142.339	0.113
Graduation	−0.697	0.498	0.194	1.282	0.148
Schober	−0.234	0.792	0.522	1.202	0.273

*OR* odds ratio, *RF* rectus femoris muscle flexibility, Hand-grip-strength, *Dhg* dominant hand-grip-strength, Hand-grip-strength, *NDhg*, non-dominant hand-grip-strength; ^*^ *p* < 0.05

Among adults, graduation level was more associated with sports injury occurrence (*p* = 0.031); the binary logistic regression model showed a statistical significance in predicting sports injury occurrence (*p* < 0.001; Table 8).

**Table 8:** Binary regression of injury risk factors in Brazilian jiu-jitsu adult practitioners

Variable	Coefficient	OR	Border values CI (95%)		*p*-value
Constant	−9.459	–	–	–	0.761
Age	−0.075	0.928	0.621	1.386	0.716
Female sex	−1.433	0.239	0.001	56.166	0.607
Height	13.156	516.869	0.000	8.491	0.318
Body weight	−0.094	0.910	0.811	1.022	0.111
Time of practice	−0.076	0.927	0.848	1.014	0.099
Exposure time	−0.015	0.985	0.696	1.396	0.934
RF, Left leg	0.082	1.085	0.941	1.252	0.262
RF, Right leg	−0.105	0.900	0.783	1.036	0.141
Hand-grip-strength, Dhg	−0.023	0.977	0.943	1.012	0.195
Hand-grip-strength, NDhg	0.027	1.027	0.986	1.070	0.204
Sit-and-Reach test	0.031	1.032	0.902	1.179	0.649
Posture 1	1.353	3.869	0.377	39.718	0.255
Posture2	0.256	1.292	0.053	31.265	0.875
Physical activity	−0.796	0.451	0.108	1.877	0.274
Graduation	−2.152	0.116	0.016	0.824	0.031*
Schober	−0.023	0.977	0.543	1.758	0.939

*OR* odds ratio, *RF* rectus femoris muscle flexibility, Hand-grip-strength, *Dhg* dominant hand-grip-strength, Hand-grip-strength, *NDhg* non-dominant hand-grip-strength; ^*^ *p* < 0.05

Differentially, a binary logistic regression model destined to present association between risk factors and sports injury occurrence in master subjects did not show statistical significance (*p* = 0.06; Table 9).

**Table 9:** Binary regression of injury risk factors in Brazilian jiu-jitsu master practitioners

Variable	Coefficient	OR	Border values CI (95%)		*p*-value
Constant	−13.067	–	–	–	0.545
Age	−0.165	0.848	0.582	1.236	0.391
Female sex	4.593	98.797	0.191	51,068.442	0.150
Height	3.134	22.954	0.000	8.628	0.756
Body weight	−0.056	0.945	0.833	1.073	0.382
Time of practice	0.039	1.040	0.995	1.087	0.085
Exposure time	0.168	1.183	0.749	1.870	0.471
RF, Left leg	0.190	1.209	0.962	1.519	0.103
RF, Right leg	−0.091	0.913	0.729	1.144	0.430
Hand-grip-strength, Dhg	−0.004	0.996	0.967	1.026	0.792
Hand-grip-strength, NDhg	−0.005	0.995	0.966	1.025	0.744
Sit-and-Reach test	0.171	1.186	0.920	1.531	0.189
Posture 1	3.417	30.464	0.715	1298.46	0.074
Posture2	−5.184	0.006	0.000	0.797	0.040*
Physical activity	−2.617	0.073	0.000	16.477	0.344
Graduation	−0.336	0.714	0.171	2.980	0.644
Schober	0.438	1.550	0.772	3.115	0.218

*OR* odds ratio, *RF* rectus femoris muscle flexibility, Hand-grip-strength, *Dhg* dominant hand-grip-strength, Hand-grip-strength, *NDhg* non-dominant hand-grip-strength; ^*^ *p* < 0.05

## Discussion

As main results of the present study, the Masters group showed a higher occurrence of injuries as well as musculoskeletal changes than other age groups. In general, age and global flexibility constituted the more directly associated factors with sports injuries onset in the present casuistry. Notably, other factors have been evidenced as predictive factors for sports injuries within each age group, including sex and exposure time for adolescents, and graduation for adults practitioners of Brazilian jiu-jitsu.

The occurrence of sports injuries and their associations with internal and external characteristics in Brazilian jiu-jitsu subjects are poorly studied, and current investigations have not described the profile of these factors [28, 29]. In the present study, Masters group showed a higher occurrence of injuries as well as musculoskeletal changes than other age groups. Also, it is probable that different indexes of sports injuries occurrence among age groups (Table 2) have been associated with multifactorial clinical aspects, including anthropometrical features and time of practice. Anthropometrical differences may result from dietary conduct combined with age development and training practice [30]. Other possible internal characteristics were investigated, such as muscle flexibility, back mobility, physical activity level and maximal isometric handgrip strength. However, potential associations between these diverse factors and sports injury onset were not detected in master subjects (Table 9).

Concerning the physical activity level, this variable is commonly used in studies for specific population groups [17]. To verify whether regular physical activity affects training performance, IPAQ has been administered in studies on sports sciences. In general, Brazilian jiu-jitsu constitutes the primary sporting modality of the participants of the present study. According to IPAQ results, high intensities of training are commonly used to improve athletic performance in Brazilian jiu-jitsu, impacting on physical activity levels. Concerning muscle strength, measures of maximal isometric handgrip strength are affected by age and sex, since increased age values have been associated with higher strength responses, despite possible normalizations by other variables, such as body weight [27]. The results presented confirmed this supposition (Tables 1 and 2).

Flexibility and adequate joint mobility are very common characteristics among practitioners subjects of Brazilian jiu-jitsu, since training practice and combat circumstances are accompanied by activities of muscle stretching [31]. Therefore, adequate back mobility and muscle flexibility may result from sports practice of Brazilian jiu-jitsu (Table 3). Regression analysis sustained that disturbed flexibility of the posterior muscle chain (OR = 1.189; *p* = 0.004) is associated with sports injury occurrence in Brazilian jiu-jitsu practitioners. Importantly, Master subjects showed increased prevalence of sports injuries and muscle retraction in global context (Tables 2, 3, 4).

Practice of combat modalities is characterized by important biomechanical requirements for lower limbs in order to ensure static and dynamic motor control during fights [1, 2, 4]. As a result, increased and specific musculoskeletal demands can be established, with potential possibilities for development of muscle retractions and posture alterations. Since muscle flexibility is also affected by age [12], Brazilian jiu-jitsu demands along with age-induced effects may explain the lower posterior muscle chain flexibility in adult and Master participants.

Intriguingly, results evidenced that the Adolescents has presented higher weekly time exposure of training than the other age groups (Table 2). Besides exposure time, female sex has also emerged as the most directly associated internal factors for sports injury occurrence in Adolescents, although this group has revealed higher proportion of female participants (Table 1). In order to increase possibilities of sport success in the adult phase, sports training schedules for infant and adolescent practitioners have tried to hasten improvements in physical and technical performance [32]. However, inadequate training planning can promote excessive time of exposure and physical overload, which can impair motor development, with potential risks for sports injury occurrence in Adolescent subjects [32, 33]. Despite the increased weekly training, it’s possible that Adolescents group has showed impaired physical and technical performance, since *tumbling/ trauma* constituted the main etiological circumstance for onset of sports injuries in Adolescent practitioners (Table 4). These mechanisms are the main and most common conditions associated with different situations of attack and defense during Brazilian jiu-jitsu practice [2–4]. Also, shoulders constituted the most frequently injured anatomical site, and *tumbling/ trauma* were the main etiological circumstances for this (Table 5).

Moreover, female sex constituted another predictive factor for the occurrence of sports injuries in adolescent practitioners. Authors postulated that the female-athlete triad is the main factor responsible for the increased female susceptibility to sports injuries in comparison to male subjects [10]. This condition is characterized by increased physical fatigue associated with nutritional disorders, menstrual disturbances, and lower bone density. Other characteristics, such as anthropometrical measures and musculoskeletal development, have also been identified as responsible for the higher female susceptibility to sports injuries [12, 13]. Therefore, the combination between female sex and high exposure time to training has been sustaining a probable predictive mechanism for sports injury occurrence among adolescent practitioners of Brazilian jiu-jitsu.

In general, physical and technical improvements in response to Brazilian jiu-jitsu training have been accompanied by important sport competitiveness and progressive overload [1–5, 8]. On the other hand, Brazilian jiu-jitsu practice is not scheduled according to a training periodization, as observed in other competitive sports. Consequently, adult and master athletes have commonly been submitted to the high intensity of training activities not interspersed by adequate recovery periods. Accordingly, combat demands, i.e. *sweep*, were the most related mechanisms for occurrence of injuries by Adults and Masters. The high competitiveness of adult and master subjects has also been sustained by the great number of injuries in handle/hands sites derived from takedown/attack circumstances (Table 5). Some authors reported joint soreness or injury in upper limbs and knees as the most prevalent musculoskeletal injuries in Brazilian jiu-jitsu [34]. Actually, joint injuries at upper and lower limbs were the most common injuries in all age groups (Table 4).

Additionally, graduation by category constituted the most associated factors to sports injury occurrence in adult subjects (Table 8). The predictive potential of the graduation by categories is documented in literature; authors [9] have verified increased prevalence of injuries with the progression of the graduation categories. This increased susceptibility of the graduated subjects may be derived from the high intensity of training and Brazilian jiu-jitsu competitiveness.

Although the present study can contribute to new information to scientific literature concerning the pathophysiology of sport injuries in Brazilian jiu-jitsu practitioners, the etiological role of the studied characteristics must be better analyzed in prospective investigations. Furthermore, other risk factors, such as biomechanical factors, and anthropometrical characteristics, should also be clarified in future studies concerning sports injury occurrence in adolescent, adult, and master Brazilian jiu-jitsu practitioners.

## Conclusion

Joint injuries in the shoulder derived from combat demands were the main sports injuries in all age categories of Brazilian jiu-jitsu. 30-year-old subjects or older presented a higher occurrence of clinical changes and retrospective musculoskeletal injuries in relation to other age groups. Female gender and exposure time constituted the main predictive factors in adolescent practitioners, while graduation category was more directly associated with retrospective injury occurrence in the adult group.

## Abbreviations

CI: 95% confidence intervals
FJJD-MS: Federação de Jiu Jitsu Desportivo do Estado de Mato Grosso do Sul, Campo Grande, MS, Brazil
IPAQ-SF: International Physical Activity Questionnaire - Short Form
One-Way ANOVA: one way analysis of variance
OR: odds ratios
